# Responses of Runoff and Soil Loss on Slopes to Land Use Management and Rainfall Characteristics in Northern China

**DOI:** 10.3390/ijerph18189583

**Published:** 2021-09-11

**Authors:** Haiyan Fang

**Affiliations:** 1 Key Laboratory of Water Cycle and Related Land Surface Processes, Institute of Geographic Sciences and Natural Resources Research, Chinese Academy of Sciences, Beijing 100101, China; fanghy@igsnrr.ac.cn; 2 College of Resources and Environment, University of Chinese Academy of Sciences, Beijing 100049, China

**Keywords:** runoff plot, soil conservation measure, runoff, soil loss, Northern China

## Abstract

Soil conservation measures are widely used to control soil erosion and sediment loss; however, their proper usage relies on a deep understanding of the responses of runoff and sediment loss to land management and rainfall characteristics. In the present study, a long-term (2014–2020) monitored dataset derived from ten runoff plots in the upstream catchment of the Miyun Reservoir in Beijing, China, was used to study runoff and sediment loss responses to land use management and rainfall characteristics. The study results show that plots with no soil conservation measures had the highest runoff depth of 75 mm and suffered the highest sediment loss, at a rate of 3200 t km^−2^ yr^−1^. The terraced and vegetated plots generated lower runoff depths, with soil loss rates less than 213.0 t km^−2^ yr^−1^. With the exception of the contour tillage plots on steep slopes, the vegetation and engineering measures can efficiently reduce runoff and sediment loss, with both runoff and sediment reduction efficiencies higher than 76%. Statistical analyses indicate that, on the plots of bare soil and cultivation without soil conservation measures, runoff and sediment loss were mainly affected by the maximum 30 min rainfall intensity. However, on the plots with soil conservation measures, they were mainly determined by rainfall amount and duration. The sediment loss rate can be well fitted with the runoff depth using a power function. Based on the analyses, water-saving soil conservation measures are recommended for the study area. In addition, the size of terraces should be reconsidered on gentle slopes, and the coverage of forest, shrubs, and grass on slopes should be reduced, thus allowing for more surface runoff generation to ensure drinking water safety. In general, for the study area, soil conservation measures are required on the bare soil and cultivated slopes.

## 1. Introduction

Soil erosion by water is a well-recognized eco-environmental problem. Erosion by water comprises the processes of runoff generation, soil detachment or entrainment, transportation of soil particles, and sediment deposition [1]. During these processes, hydrological, physiochemical, and biological factors are often attributed to soil erosion, resulting in on- and off-site environmental problems. The depletion of nutrients, soil organic material, and top soil causes land degradation and influences crop productivity. The progression of water erosion significantly affects the level of land degradation. In addition, water erosion can also cause downstream water pollution, reservoir sedimentation, river and harbor silting, and ecological and recreational impacts of sediment management [2,3,4,5], threatening the biodiversity, water resource use, and the natural ecosystem. To reduce soil loss and its negative impacts, soil conservation measures are widely used globally [6,7]. To be able to make appropriate use of land and water resources, it is vitally important to deeply understand the effects of different soil conservation measures on runoff and soil loss. Multiple methods are used to study soil erosion, including runoff and sediment measurements from runoff plots [8], rainfall simulation [9], radionuclide tracers [10], soil erosion models, and remote sensing techniques [11]. Among these approaches, the runoff plot is the most common and traditional method used to study erosion by water. Numerous soil erosion models, such as the Universal Soil Loss Equation (USLE) and the Water Erosion Prediction Project (WEPP), were established depending on runoff plots because model parameters are required to be calibrated using the measured runoff and sediment from runoff plots [12]. Furthermore, using the measured runoff and sediment from runoff plots, the effects of slope characteristics, soil conditions, rainfall characteristics, and land use and soil conservation measures on runoff, sediments, or nutrients have been widely studied, and numerous meaningful results have been derived [13,14]. However, in some places, the runoff and sediment, and their relationships, or their responses to rainfall and different soil conservation measures, are not yet well understood due to the lack of runoff plots. Furthermore, deep insights into runoff generation and soil erosion characteristics have yet to be obtained because systematic research has not been conducted.

Soil conservation measures are widely used globally to prevent soil loss and achieve sustainable development goals (SDGs), and numerous significant study findings have been published. For example, Zhao et al. [13] summarized the runoff plot data on the Chinese Loess Plateau, and found that, due to the implemented soil conservation measures, soil and water losses were reduced greatly, but not down to the background levels. Wolka et al. [14] reviewed the effect of soil conservation measures on runoff, soil loss, and crop yields in Sub-Saharan Africa, and found that the impact of soil conservation measures on crop yields varied with rainfall and slope gradient. Maetens et al. [6] also evaluated the effects of soil conservation measures in reducing runoff and soil losses from runoff plots in Europe and the Mediterranean region. Xiong et al. [15] also reviewed the effects of soil conservation measures on soil loss control at a global scale. Collectively, these results improve our understanding of the effects of soil conservation measures on soil erosion and water loss. However, most of these studies focus on the local runoff and soil loss control, and less consideration has been given to their effect on downstream water resource use. Moreover, the effects of the implementation of soil conservation measures on SDGs have been neglected at the basin scale.

Climate change will likely affect water erosion due to various factors, including vegetation growth, soil moisture, and the amount and intensity of precipitation [1]. In recent years, the soil loss rate has increased, as a result of climate and environmental changes and anthropogenic activities [1,16]. However, the impacts of soil conservation measures on runoff and soil loss due to climate change have received less attention, although some research has been undertaken globally. Therefore, systematic analysis of runoff and soil loss characteristics, and their interaction, under different rainfall properties, provides an understanding of soil erosion characteristics under climate change.

The Miyun Reservoir has a water capacity of around 4 billion m^3^ and a catchment area of 14,924 km^2^. This reservoir is one of the most important drinking water sources in Beijing [17,18,19,20], providing approximately 70% of the drinking water for millions of people in Beijing City [21]. Wang et al. [22] and Zhang et al. [23] found that the water pollution of the Miyun Reservoir was caused by serious soil erosion in its upstream catchment, and accounted for about 60% of incoming nitrogen sources. Since the 1980s, the Beijing government has greatly promoted large-scale soil conservation measures to control soil loss and reduce water pollution. Since 2000, a significant effort has been undertaken in constructing soil conservation measures, such as terracing, fish-scale pits (i.e., pits with a certain water storage capacity that are staggered on slopes and look like fish scales with a semicircular or crescent shape), level benching, and the use of contour tillage, in the upstream catchment of the Miyun Reservoir [19,20]. These measures greatly influenced the downstream water resource. For example, Li [24] demonstrated that the runoff discharge was reduced by the implemented soil conservation measures. Liu et al., [25] constructed the Chinese Soil Loss Equation (CSLE) to model the runoff and sediment loss from runoff plots in the upstream catchment of the Miyun Reservoir. Qiu et al. [19] evaluated the impacts of different best management practices on runoff, soil loss, and nutrient loss using the SWAT model.

Zhang et al, [26] and Tang et al. [27] evaluated land use management impacts on nonpoint source pollution in the upstream catchment of the Miyun Reservoir. The effect of land use management practices on river discharge was studied by Qiu et al. [19,20], who found that climate variability and land use management practices have significant effects on runoff, sediment yield, and nutrient loss. Tang et al. [27] found that land use change decreased TN and TP losses by 39.1% and 23.7%, respectively. Future climate change impacts on streamflow and nitrogen exports were predicted by Yan et al. [28]. Similar study results were also reported in the literature [29,30,31,32]. However, most of these studies were conducted at the catchment scale, and few studies have focused on the plot scale [33,34]. This has resulted in knowledge gaps regarding the effects of different soil conservation measures on runoff and soil loss, in addition to their responses to rainfall characteristics at different spatial scales.

Therefore, using ten runoff plots with different vegetation and engineering measures in the Miyun Reservoir catchment, the objectives of the present study were to (i) explore the characteristics of the runoff and soil loss rate (SLR) on slopes; (ii) understand the effect of different soil conservation measures on the runoff and SLR; and (iii) identify the relationships of the runoff and sediment, and their responses to rainfall characteristics. Finally, some implications and suggestions are provided to improve land use and water resource management in the study area.

## 2. Materials and Methods

### 2.1. Study Area

**Figure 1:**
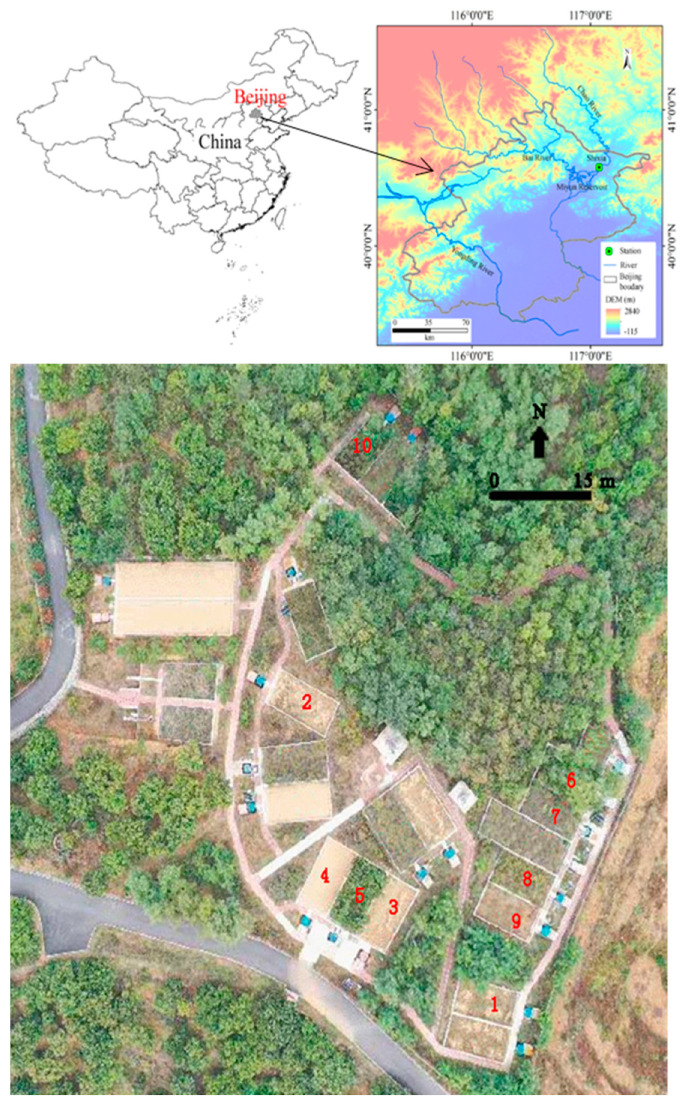
Study area and the spatial distribution of the runoff plots. The red numbers represent runoff plots.

The runoff plots were located in the Shixia catchment (E117°4′30″ and 40°34′40″ N) upstream of the Miyun Reservoir, which is about 40 km northwest of Beijing (Figure 1). The catchment covers an area of 33 km^2^. The study region has a temperate territorial monsoon climate, with annual precipitation ranging from 331 to 615 mm, based on climate data from 2007 to 2020, 70% of which falls from June to August. The catchment elevation ranges from 130 to 390 m a.s.l. The main lithology is gneiss, scattered with granite and limestone. The runoff plots have an eluvial cinnamon soil with soil depths around 30 cm.

The main vegetation types are Robinia pseudoacacia, Pinus tabulaeformis, and Chestnut forest plantation, and the main crops are corn and wheat. The sandy loam soil contains particles with the following size distibution: around 60.9% larger than 0.05 mm in diameter, 18.22% with a diameter of 0.05–0.005 mm, 9.22% with a diameter of 0.005–0.001 mm, and 14.88% with a diamter of less than 0.001 mm [22].

### 2.2. Data Collection

In the study area, ten runoff plots were used. Each plot had an area of 50 m^2^ and a length of 10 m. The boundary of each plot was constructed of bricks and cement, and extended 30 cm above the ground and 40 cm into the soil to prevent runoff from leaving or entering the plots. The slopes of the plots ranged from 3.5° to 27°. The conservation measures implemented on the plots included contour tillage, terracing, and level benching, and vegetation measures such as grass, shrubs, and forest. Corn was planted on the cultivated plots. Detailed information is given in Table 1.

From 2014 to 2020, runoff and sediment discharges from the plots were collected with a nine hole diversion bucket and a tank at the end of each runoff plot. After each rainfall event, the runoff amount was measured. Evenly mixed water and sediment samples were also collected with 1000 mL flasks, and transported to a laboratory where sediment concentrations were determined based on the ratio of dry sediment mass and the runoff-sediment volume (i.e., gravimetric method) (kg m^−3^). Runoff depth (H; mm) of each rainfall event was calculated using rainfall amount and plot area. SLR (t km^−2^ event^−1^) was also calculated using runoff amount, sediment concentration, and plot area.

(1) $$H=1000\times \frac{\mathrm{TR}}{A}$$

(2) $$\mathrm{SLR}=1000\times \frac{\mathrm{TR}\times \mathrm{SC}}{A}$$

where TR represents total runoff amount (m^3^), SC represents sediment concentration, and A is runoff plot area (m^2^). Annual H and annual SLR were obtained by summing their respective event values for each plot.

Rainfall information was recorded by a rain gauge and a rain barrel near the plots. In the present study, an erosive rainfall was defined as one that induced erosion on any of the plots. Flows may be present on some plots but absent on other plots due to different soil infiltration capacities of the plots, as proposed by Zhu and Zhu [35]. For each erosive rainfall event, five rainfall eigenvalues, namely, rainfall duration (RD), rainfall amount (P), mean rainfall intensity (I_m_), and maximum intensities at 30 min (I_30_) and 60 min (I_60_) were obtained.

### 2.3. Data Treatment and Statistical Analysis

Annual runoff and soil loss control efficiencies of the plots were calculated based on the values of annual H and SLR on bare plot #4.

(3) $$\mathrm{CE}=100\times \frac{X_{0}-X_{i}}{X_{0}}$$

where CE represents annual H or annual soil loss control efficiency, X_0_ represents annual H or annual SLR on plot #4, and X_i_ represents the corresponding H or SLR values on other plots (i = 1, 2, 3, 5, 6, …, 10).

In the study area, Liu [25] derived a formula to calculate rainfall erosivity using event P and the corresponding I_30_:

R = 0.2463 × P × I_30_ (4)

where R (mm × mm h^−1^) is rainfall erosivity, and P and I_30_ are rainfall amount (mm) and maximum 30 min rainfall intensity (mm h^−1^), respectively.

To study the impact of rainfall properties on runoff generation and soil loss, Pearson correlation and multiple regression analyses were used to evaluate the relationships between H, SLR, and the five rainfall properties (i.e., RD, P, I_m_, I_30_, and I_60_). Fisher’s protected least significant difference test was also used to compare the means of Hs and SLRs. Treatments were considered significantly if *p*-value < 0.05. All the statistical analyses were conducted using SPSS version 14.0 for Windows.

## 3. Results and Discussion

### 3.1. Rainfall Characteristics

For the 66 erosive rainfall events in 2014–2020, RD ranged from 20 to 1940 min, with an average of 411 min and a standard error (Std. E) of 51.44 mm (Table 2). Erosive rainfall amounts ranged from 4.8 to 108 mm with an average of 31.5 mm. The values of I_m_ ranged from 1.03 to 54.0 mm h^−1^, with an average of 104.5 mm h^−1^. In comparison, the values of I_30_ and I_60_ were much higher. The largest I_30_ reached 64.20 mm h^−1^. In comparison, the largest I_60_ was 61.70 mm h^−1^ with a standard error of 1.46 mm h^−1^. Rainfall erosivity R ranged from 7.09 mm × mm h^−1^ to 1491 mm × mm h^−1^, with an average of 234.83 mm × mm h^−1^.

Each of these five eigenvalues varied greatly from year to year during the study period. The mean values of RD were 180.63 min in 2014, 271.03 min in 2015, 425.00 min in 2016, 426.71 min in 2017, 694.00 min in 2018, 365.27 min in 2019, and 47.78 min in 2020. The annual values of I_30_ were 21.00 mm h^−1^ in 2014, 25.78 mm h^−1^ in 2015, 26.15 mm h^−1^ in 2016, 31.31 mm h^−1^ in 2017, 22.92 mm h^−1^ in 2018, 32.19 mm h^−1^ in 2019, and 22.91 mm h^−1^ in 2020. Mean event P fluctuated around a depth of 30 mm from 2014 to 2020, with a higher coefficient of variation. The mean values of event rainfall erosivity also varied greatly, with larger values in the years 2017–2019, and lower values in other years.

### 3.2. Runoff Depth and Soil Loss Rate of the Runoff Plots

The minimum rainfall amount required to generate runoff (i.e., the threshold rainfall amount 4.8 mm), the number of rainfall-runoff events, H, and SLR varied significantly among the plots (Table 3). From 2014 to 2020, the total number of rainfall-runoff events per year varied from zero to 62. The bare and cultivated plots had higher H and SLR than those plots with soil conservation measures. Annual values of H decreased from 75.3 mm on plot #4 to zero on plot #10, and annual values of SLR decreased from 3205 t km^−2^ yr^−1^ on plot #3 to zero on plot #10. At the event scale, both H and SLR had similar patterns to those at the annual scale. Only small amounts of runoff and soil loss occurred on the plots with soil conservation measures, with the exception of plot #2. These SLR values were significantly lower than the tolerable threshold of 200 t km^−2^ yr^−1^ in the study area [36] and the global mean tolerable value, which ranges from 0.1 to 1 mm yr^−1^ [37].

**Figure 2:**
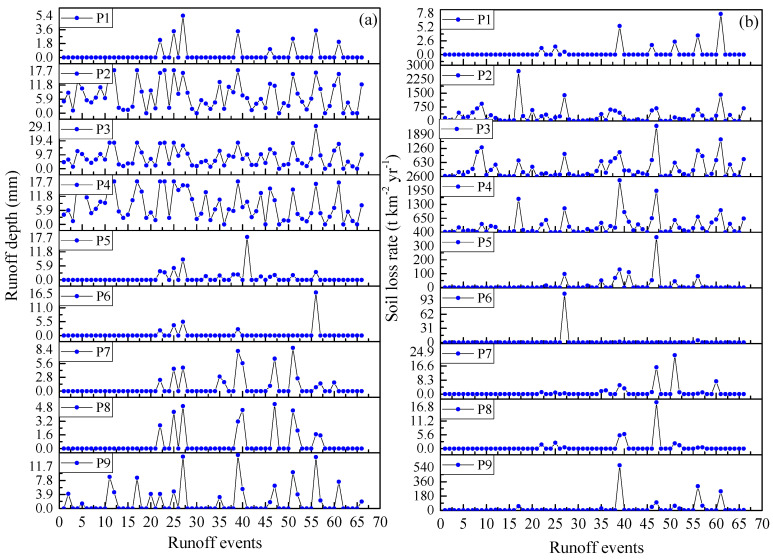
Temporal changes of runoff depth (**a**) and soil loss rate (**b**) during 2014–2020.

At an event scale, temporal changes of soil erosion and runoff are shown in Figure 2. In respect of runoff depth, the highest runoff depths were 18.08 mm on plot #2, 29.65 mm on plot #3, and 18.25 mm on plot #4. Lower runoff depth occurred on plots #7–#9, with the largest being 8.75, 5.21, and 14.92 mm. The runoff depth on plots #1, #5, and #6 were the lowest. When runoff events occurred on plots #1, #5, and #6, runoff events also occurred on other plots (Figure 2). During 2014–2020, runoff depths on plots #2–#4 decreased slightly from 2014 to 2020, whereas there were no changing trends for the runoff depths on other plots. Regarding the soil loss rates on the plots, the temporal trend did not occur for all the plots. The change amplitude was larger than that of runoff plot for each plot. For example, the soil loss rate on plot #3 changed from 0.47 to 2278 t km^−2^ yr^−1^ as the runoff depth changed from around 0 to 29.65 mm.

### 3.3. Impact of Soil Conservation Measures

**Figure 3:**
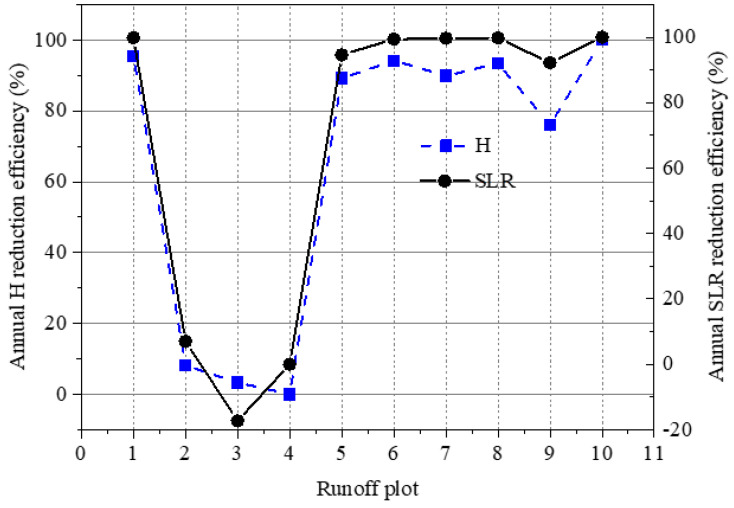
Reduction efficiencies of annual runoff depth (H) and annual soil loss rate (SLR) in 2014–2020.

**Figure 4:**
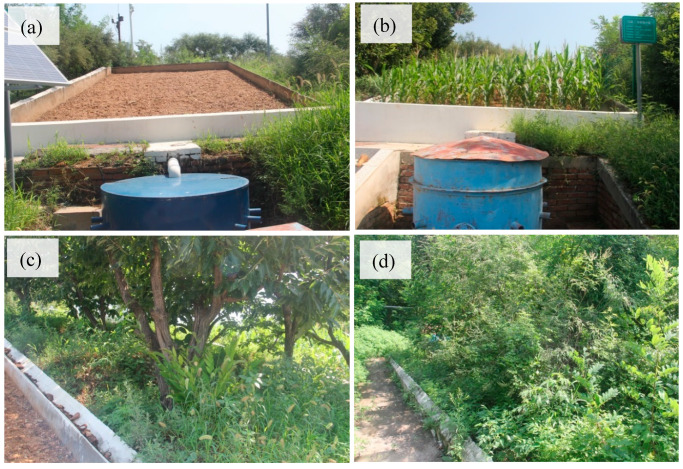
Photos taken at the experimental station: (**a**) bare plot, (**b**) cultivated plot, (**c**) Chestnut plot, and (**d**) forest plot.

In the study area, vegetation measures, such as planting forest (i.e., plot #6), shrubs (i.e., plot #7), and grass (i.e., plots #8 and #9), in addition to their combinations (i.e., plot #10), significantly reduced H and SLR (*p*-value < 0.01). In comparison to plot #4 of bare soil, annual H reduction efficiencies ranged from 3.32% to 100%, and annual SLR reduction efficiencies ranged from −17.30% to 100% (Figure 3). The H and SLR reduction efficiencies of plots #2 and #3 were much lower, even with a negative value for plot #3. This is because the higher stone content in the topsoil of the plot #4 increased surface roughness (Figure 4) and resulted in a lower SLR than that from plot #3. This phenomenon has been reported in the literature [38,39]. In comparison, over 75% of H and SLR reduction efficiencies occurred on the plots #1, #5–#10 (Figure 3). This can relate to the impact of vegetation measures on hydrological and soil erosion processes through intercepting rainfall, altering runoff infiltration into soils, and changing runoff and sediment flow velocity on the ground [40,41]. In contrast to the mono-species communities, stratified vegetation communities can effectively reduce runoff and soil loss [36,42]. Due to dense forest, grass, and shrub coverage, no runoff occurred on plot #10 from 2014 to 2020, despite having a steep slope (Table 1). The runoff and SLR reduction efficiencies were comparable to the reported results from the literature [43,44,45]. As expected, high-coverage grass was more efficient in reducing H and SLR than low-coverage grass. Due to the dense surface cover, the shrub and grass plots #8 and #9 were better able to control soil loss than the forest plot #6, because lower vegetation is more efficient in reducing rainfall splash erosion, increasing water infiltration into the soil, and controlling soil loss [46,47,48].

In recent years, due to the reduced precipitation and increased implementation of management practices, decreased surface runoff and sediment loss have been reported in literature [24,28,46,49,50]. The reduction of runoff induced by soil conservation measures on slopes has led to the decreased water discharge into the Miyun Reservoir [19,20]. As a result of the implemented soil conservation measures, more runoff was intercepted and infiltrated into the soils, and thus made available for human use through wells, or eventually by feeding the Miyun Reservoir through groundwater seepage. However, the intercepted runoff can also be used by vegetation, and then lost through evapotranspiration [51]. The effect of forest or reforestation on runoff is a highly complex issue, and is beyond the scope of the present study. However, in the study region, research found that the time at which soil conservation measures were widely implemented coincided well with the decrease in water discharge into the downstream reservoir [52]. This implies that the implementation of soil conservation measures reduced the downstream water resource. Therefore, the strategies for implementation of soil conservation measures should be adjusted to allow more water discharge to the reservoir to ensure drinking water safety for Beijing city [19,20]. The current research shows that lower vegetation coverage can also decrease SLR to below the tolerable value (i.e., 200 t km^−2^ yr^−1^) and allows for more runoff generation in the study area. For example, annual SLR values on plots #6–#8 in Table 3 were considerably lower than the tolerable level and their annual H values were less than 8 mm due to dense vegetation cover (Table 1). Therefore, the vegetation coverage should be lower. Similarly, the terrace plot (i.e., plot #1) intercepted around 95.5% of water on the gentle slopes (Figure 3). The terracing practices should not be implemented on gentle slopes, or the terrace size should be adjusted to allow for more runoff generation.

Chestnut forests are widespread in the study area, particularly on steep slopes. Because weeds under chestnut trees are often completely cleared by hand, without leaving stalks or residue, the SLR on the 8°–25° slopes was higher than 2430 t km^−2^ yr^−1^ [53], which significantly exceeds the threshold tolerable value of 200 t km^−2^ yr^−1^. Due to the completely exposed soil surface, higher soil erodibility, and less biological diversity, serious soil erosion in forested areas was also reported in previous literature [54,55,56]. For example, Wang et al. [55] found that rills and ephemeral gullies developed under *Pinus massoniana* trees, where the soil layer depth of 71.2 mm was eroded away. Yuan et al. [56] also undertook a systematic review of soil loss under economic forests. In the present study, because the soil surface of the chestnut forest was covered with weeds on a level bench (Figure 4c), plot #5, which had a steep slope, had a larger threshold rainfall amount and a much smaller SLR value (i.e., 147.9 t km^−2^ yr^−1^) than other plots, and there were only eight erosive rainfall events from 2014 to 2020. Therefore, soil surface protection should be considered in the chestnut forest lands of the study area. Level benching and/or other soil conservation measures may better control soil losses in chestnut forests.

In the present study, plots #5–#8 had comparable slopes and H values, whereas plots #5 and #6 with engineering measures produced higher SLR values than those from plots #7 and #8 with vegetation measures. This is because the vegetation measures are more efficient in controlling soil loss through reducing sediment concentration [44]. In contrast, the engineering measures, such as terracing, level benching, and fish-scale pits, can decrease slope gradient and slope length, and change the topographic characteristics, Thus, they are more efficient in intercepting runoff, resulting in higher threshold rainfall amounts (Table 3). The combined use of vegetation and engineering soil control measures can better control soil loss in the study area.

### 3.4. Impact of Rainfall on Runoff and Soil Loss

The Pearson correlation coefficient matrix in Table 4 indicates that among the five rainfall eigenvalues (i.e., P, RD, I_m_, I_30_, and I_60_), only P and I_30_ were significantly correlated with H for all the plots, whereas the mean rainfall intensities (i.e., I_m_) were not significantly correlated with H for all the plots. In contrast, with the exception of I_m_, all of these rainfall eigenvalues were significantly correlated with H on the cultivated and bare soil plots #1–#4. The RD values were significantly correlated with H on the plots #1–#6 at 0.05 and/or 0.0 levels, and not significantly correlated with other plots’ vegetation measures. This indicates that the vegetation measures are less efficient in intercepting runoff than the engineering measures. The correlation coefficients were much lower for the plots covered with both vegetation and engineering measures [45].

Similarly, P and/or I_30_ were significantly correlated with SLRs on the plots #1–#7 and #9 and not significantly correlated on the plot #8, resulting from dense grass cover on the soil surface. I_60_ was only significantly correlated with SLR for the cultivated and bare plots #1–#4, and the mean rainfall intensities (i.e., I_m_) were insignificantly correlated with SLR values for all the plots. No significant correlations existed between RD and SLR values, with the exception of plot #6, which was covered with forest (Table 1).

The stepwise regression functions in Table 5 indicate that H can be significantly determined by P, I_30_, and other rainfall eigenvalues, with a determinant coefficient R^2^ higher than 0.40 for the cultivated and bare soil plots (i.e., plots #1–#4). H was significantly influenced by P and/or I_30_ on all the plots. The soil conservation measures greatly influenced their relationships [57], resulting in lower R^2^ on the vegetated plots. H was not correlated with I_30_ on plots #1, #5, and #6 with engineering measures. On these plots, mean annual H was only 3.4 mm, which was much less than on most of the other plots. Cai [58] and Xiao et al. [59] reported that little soil loss occurred on terraced land when event P was less than 50 mm, which is higher than the event-averaged value in the present study (i.e., 31.51 mm; Table 2).

In contrast to the regression relationships between H and the rainfall eigenvalues, SLR was linearly correlated with I_30_ on most plots (Table 6). On the plots #1, #6, and #9, P and RD significantly influenced SLR. Impacted by high-coverage grass, no rainfall eigenvalues were selected by the regression function for plot #8. The intensive vegetation measures also reduced the impact of rainfall intensity on SLR. A similar conclusion was also reported by Liu et al. [60]. 

### 3.5. Relationships between H and SLR

There were positive relationships between H and SLR for all the plots (Table 7). In comparison to the linear functions, the power functions (y = ax^b^) were better able to describe their relationships and had higher R^2^ values. This kind of regression function was also widely reported in the literature [61,62]. Physically, the exponent b in the power function reflects the sensitivity of the soil surface to rainfall. Higher b values appeared for the bare soil and cultivated plots, with the exception of the terraced plot #1, and smaller b values occurred for the plots with intensive vegetative or engineering measures. The finding of a smaller b value for plot #8 than for plot #9 further proved that vegetation can decrease SLR by reducing surface runoff and sediment concentration [63]. Notably, the b value for plot #2 was comparable to those for bare soil and cultivated plots, further implying that contour tillage on steep slopes cannot effectively reduce soil loss. Similar findings were also reported in other regions [13,44]. Therefore, alternative soil conservation measures should be considered for cultivated slopes having steep gradients.

### 3.6. Multivariate Analysis of Slope Degree, Soil Cover, and Rainfall Erosivity

Rainfall erosivity reflects the properties of event P and rainfall intensity. Therefore, slope degree, soil cover, rainfall duration, and rainfall erosivity were used to model their impacts on soil loss. The GLM model provided a result with an adjusted R^2^ of 0.37. The partial ŋ^2^ indicated that soil cover was the most important factor, followed by rainfall erosivity and slope degree (Table 8). A similar contribution was also identified in Syria by Mohammed et al. [64], where the partial ŋ^2^ of land cover was 0.132. The effect of the interaction of slope gradient and land use was not significant in influencing soil loss, which can be explained by their complex function in reducing soil erosion (Table 1). Slope steepness had a positive effect on soil loss, and dense vegetation cover negatively influenced soil loss.

## 4. Conclusions

In the study area, with the exception of contour tillage on steep slopes, all of the soil conservation measures were found to effectively reduce runoff and soil loss, with runoff and soil loss reduction efficiencies greater than 76%. The use of contour tillage on steep slopes cannot effectively control soil loss, and the bare soil and cultivated slopes without any soil conservation measures suffered from serious soil loss, with SLR values higher than 3200 t km^−2^ yr^−1^. Vegetation measures can control soil loss by reducing surface runoff and sediment concentration, whereas engineering measures can control soil loss mainly by intercepting surface runoff. The combined usage of vegetation and engineering measures can provide better soil loss control.

As a result of soil conservation measures, the relationships between H, SLR, and the rainfall eigenvalues varied significantly between plots. The H and SLR values on the plots with no soil conservation measures were mainly affected by rainfall intensity (i.e., I_30_), whereas on the plots with soil conservation measures, they were mainly controlled by P and RD. The SLR value of each plot was able to be better fitted with H using a power function than a linear function. In the study area, land use and rainfall were found to be the two factors controlling soil loss.

For the study area, and similar areas globally, the tradeoff between soil loss control and drinking water safety should be considered and evaluated thoroughly when soil conservation measures are implemented. In general, soil conservation measures are required on bare soil and cultivated slopes to reduce soil loss. In the study region, contour tillage on steep slopes was not sufficiently effective, having an annual SLR reduction efficiency of less than 20%. Thus, the combination with other measures can be considered. However, terracing, fish-scale pits, and level benching measures should be considered on steep slopes. The size of terraces should be reconsidered on different slopes. The density of vegetation coverage should be reduced to a certain value to ensure that the obtained SLR is just under the tolerable value, which can thus allow for more runoff. Overall, for the safety of downstream drinking water, water-saving soil conservation measures are preferred in the study area.

## Figures and Tables

**Table 1:** Basic characteristics of the 10 runoff plots in the present study.

Plot #	Land Use	Gradient (°)	Soil Depth (cm)	Vegetation	Vegetation Coverage (%)	Measure
1	Cultivated	3.5	60	Corn		Terrace (width: 4 m)
2	Cultivated	14.4	20	Corn		Contour tillage
3	Cultivated	16.5	30	Corn		-
4	Bare land	16.5	30	-	-	-
5	Forest	16.5	30	Chestnut (*Castanea sativa Mill.*)	50%	Level bench (width: 3 m)
6	Forest	17.1	15	Acacia (*Robinia pseudoacacia*) and Arborvitae (*Platycladus orientails L.*)	80%	Fish-scale pit (Radius: 0.5 m)
7	Shrub	18.6	15	Wattle (*Vitex negundo var.* heterophylla)	50%	-
8	Grassland	19.0	15	Alfalfa (*Medicago sativa*)	80%	-
9	Grassland	19.0	15	Alfalfa (*Medicago sativa*)	<30%	-
10	Forest, Shrub, and Grass	27.0	20	Acacia (*Robinia pseudoacacia*), Wattle (*Vitex negundo var.* heterophylla), and weeds	>95%	-

Note: vegetation coverage was visually estimated. The coverage of corn is not given because it changed rapidly during its growing period.

**Table 2:** Statistical characteristics of the five rainfall eigenvalues and rainfall erosivity for the 66 erosive rainfall events in 2014–2020.

		RD (h)	*p* (mm)	I_m_ (mm h^−1^)	I_30_ (mm h^−1^)	I_60_ (mm h^−1^)	R (mm × mm h^−1^)
2014	Mean	180.63	31.83	12.83	21.00	24.96	229.20
	Std. E	61.85	8.89	2.24	4.99	6.75	112.20
	Min.	50.00	11.80	4.77	8.50	8.50	32.33
	Max.	580.00	86.50	23.05	43.80	61.70	933.16
2015	Mean	271.00	25.41	10.44	25.78	17.51	180.58
	Std. E	94.22	8.13	3.60	4.38	3.59	64.88
	Min.	21.00	4.80	1.56	6.00	3.60	7.09
	Max.	1060.00	102.80	44.00	54.00	44.00	749.46
2016	Mean	425.00	33.16	12.51	26.15	20.37	212.74
	Std. E	168.56	8.55	3.63	3.37	2.74	53.31
	Min.	20.00	10.30	1.03	9.20	4.60	24.02
	Max.	1615.00	82.50	30.90	42.00	30.90	493.70
2017	Mean	426.71	35.61	7.42	31.31	20.60	292.74
	Std. E	150.07	8.78	1.47	5.09	2.43	82.09
	Min.	57.00	10.20	3.15	18.60	9.60	46.73
	Max.	1020.00	76.70	13.58	54.00	28.90	642.30
2018	Mean	694.00	39.52	4.33	22.93	16.13	308.64
	Std. E	175.24	10.12	0.82	4.48	3.20	132.18
	Min.	160.00	10.00	1.30	6.00	4.50	14.78
	Max.	1940.00	108.10	9.50	56.00	42.00	1491.00
2019	Mean	365.27	29.46	12.05	32.19	22.30	260.26
	Std. E	87.93	4.19	4.68	6.03	4.53	68.34
	Min.	22.00	12.10	1.08	12.00	9.10	42.92
	Max.	979.00	46.80	54.00	64.20	54.00	740.02
2020	Mean	470.78	26.58	7.16	22.91	16.46	161.92
	Std. E	137.56	5.53	2.77	2.54	2.42	40.68
	Min.	32.00	8.40	2.46	12.80	8.30	34.34
	Max.	1082.00	55.50	27.94	37.60	29.80	363.61
All	Mean	411.02	31.51	9.50	26.04	19.55	234.83
	Std. E	51.44	2.94	1.22	1.75	1.46	31.97
	Min.	20.00	4.80	1.03	6.00	3.60	7.09
	Max.	1940.00	108.10	54.00	64.20	61.70	1491.00

Notes: Std. E = standard error, CV = coefficient of variation, *p* = rainfall amount, I_m_ = mean rainfall intensity, I_30_ = maximum 30 min rainfall intensity, I_60_ = maximum 60 min rainfall intensity, and R = rainfall erosivity.

**Table 3:** Characteristics of runoff depth (H) and soil loss rate (SLR) on the 10 runoff plots at annual and event scales in 2014–2020.

Plot #	Land Use	Threshold Rainfall Amount (mm)	Outflow Number	Annual Scale (Mean + Std.)		Event Scale (Mean + Std.)	
				H	SLR	H	SLR
1	Cultivated	28.7	8	3.4 (4.1b)	3.5 (3.5b)	2.9 (1.3b)	3.0 (2.4b)
2	Cultivated	8.4	59	69.2 (19.2a)	2541.7 (893.3a)	8.2 (5.6a)	301.6 (445.3a)
3	Cultivated	4.8	62	72.8 (22.2a)	3205.2 (1116.1a)	8.2 (6.1a)	361.9 (461.9a)
4	Bare land	4.8	60	75.3 (31.5a)	2732.5 (2047.1a)	8.8 (5.9a)	318.8 (476.2a)
5	Forest	21.8	14	8.1 (10.4b)	147.9 (234.0b)	4.0 (4.5b)	73.9 (93.7b)
6	Forest	55.0	5	4.4 (7.0b)	16.3 (40.5b)	6.2 (6.2ab)	22.8 (47.2b)
7	Shrub	15.1	14	7.6 (8.2b)	9.3 (11.4b)	3.8 (2.6b)	4.7 (6.8b)
8	Grassland	15.1	10	5.0 (6.2b)	5.6 (11.0b)	3.5 (1.4b)	3.9 (5.6b)
9	Grassland	15.1	20	18.0 (11.6b)	213.2 (272.3b)	6.3 (4.3ab)	74.6 (142.0b)
10	Forest, shrub, and grass	0.0	0	0.0 (0.0c)	0.0 (0.0c)	0.0 (0.0c)	0.0 (0.0b)

Note: Average values following the numbers with the same letter in the same column are not significantly different at *p*-value = 0.05 as determined by Fisher’ protected least significant difference test. Std. = standard deviation.

**Table 4:** Pearson correlation coefficients between rainfall eigenvalues (i.e., P, I_30_, I_60_, I_m_) and runoff, and soil loss rate for the selected runoff plots in 2014–2020.

	Plot #	RD	*p*	I_m_	I_30_	I_60_
H	1	0.399 **	0.656 **	−0.019	0.330 **	0.294 *
	2	0.263 *	0.566 **	0.117	0.651 **	0.460 **
	3	0.293 *	0.642 **	0.058	0.546 **	0.407 **
	4	0.379 **	0.601 **	0.008	0.473**	0.368 **
	5	0.344 **	0.412 **	−0.117	0.212	0.146
	6	0.248 *	0.574 **	−0.020	0.122	0.148
	7	0.070	0.260 *	0.198	0.398 **	0.259 *
	8	0.196	0.359 **	0.195	0.365 **	0.250 *
	9	0.181	0.537 **	0.171	0.514 **	0.410 **
SLR	1	0.004	0.359 **	0.126	0.218	0.304*
	2	0.082	0.347 **	0.168	0.441 **	0.335 **
	3	0.002	0.310 *	0.188	0.431 **	0.317 **
	4	−0.014	0.298 *	0.223	0.410 **	0.366 **
	5	0.051	0.229	0.089	0.283 **	0.151
	6	0.460 **	0.420 **	−0.077	0.265 *	0.240
	7	−0.154	0.000	0.210	0.319 *	0.155
	8	−0.096	0.024	0.225	0.205	0.097
	9	−0.052	0.306 *	0.113	0.179	0.225

Note: Pearson correlation coefficients were not calculated for plot #10 because no runoff occurred during the study period. RD = rainfall duration, CV = coefficient of variation, *p* = rainfall amount, I_m_ = mean rainfall intensity, I_30_ = maximum 30 min rainfall intensity, and I_60_ = maximum 60 min rainfall intensity. * represents significance at 0.05 level, and ** represents significance at the 0.01 level.

**Table 5:** Multiple regression analysis for runoff depth (H) and the five rainfall eigenvalues of rainfall amount (P), rainfall duration (RD), mean rainfall intensity (I_m_), maximum 30 min rainfall intensity (I_30_), and maximum 60min rainfall intensity (I_60_).

Plot #	Regression Function	R^2^	*F*	Sig	sigP	sigI_30_	sigI_60_	sigI_m_	sigRD
1	H = 0.03P − 0.56	0.43	48.44	**	**	-	-	-	-
2	H = 0.10P + 0.31I_30_ − 0.16I_60_ − 1.05	0.57	27.45	**	**	**	*	-	-
3	H = 0.13P + 0.15I_30_ − 0.45	0.51	32.93	**	**	**	-	-	-
4	H = 0.13P + 0.12I_30_ + 0.83	0.43	23.32	**	**	**	-	-	-
5	H = 0.05P − 0.56	0.17	13.11	**	**	-	-	-	-
6	H = 0.11P-0.003RD + 0.07I_60_ − 0.34	0.45	17.01	**	**	-	*	-	**
7	H = 0.06I_30_ − 0.66	0.16	12.02	**	-	**	-	-	-
8	H = 0.01P + 0.03I_30_ − 0.60	0.19	7.24	**	*	*	-	-	-
9	H = 0.06P + 0.09I_30_ − 2.53	0.39	25.92	**	**	**	-	-	-

Note: Regression analysis was excluded; R = complex correlation coefficient, sig = significance level, sigP = the significance level of H affected by P, and sigI_30_ = the significance level of H affected by I_30_. * = significance at the 0.05 level, and ** = significance at the 0.01 level.

**Table 6:** Multiple regression analysis for soil loss rate (SLR) and the five rainfall eigenvalues of rainfall amount (P), rainfall duration (RD), mean rainfall intensity (I_m_), maximum 30 min rainfall intensity (I_30_), and maximum 60 min rainfall intensity (I_60_).

Plot #	Regression Function	R^2^	*F*	sig	sigP	sigI_30_	sigI_60_	sigI_m_	sigRD
1	SLR =−0.002RD + 0.04P − 0.20	0.25	10.57	**	**	-	-	-	**
2	SLR = 13.51I_30_ − 88.41	0.19	15.43	**	-	**	-	-	-
3	SLR = 13.97I_30_ − 30.40	0.19	14.61	**	-	**	-	-	-
4	SLR = 13.51I_30_ − 68.25	0.17	12.95	**	-	**	-	-	-
5	SLR = 1.04I_30_ − 11.95	0.08	5.57	*	-	*	-	-	-
6	SLR = 0.02RD + 0.26I_30_ − 11.19	0.29	17.19	**	-	*	-	-	**
7	SLR = 0.08I_30_ − 1.16	0.10	7.24	**	-	**	-	-	-
8	-	-	-	-	-	-	-	-	-
9	SLR = −0.11RD + 2.39P − 8.87	0.24	9.70	**	**	-	-	-	**

Note: Regression analysis was excluded; R^2^ = determinant coefficient, sig = significance level, sigP = the significance level of SLR affected by P, and sigI_30_ = the significance level of SLR affected by I_30_. * = significance at the 0.05 level, and ** = significance at the 0.01 level.

**Table 7:** Relationships between runoff depth (H) and soil loss rate (SLR) from the plots for the 66 erosive rainfall events.

Plot #	Linear Function: y = a + bx		Power Function: y = ax^b^	
	Function	R^2^	Function	R^2^
1	SLR = 0.78H + 0.09	0.42	SLR = 0.84 H^0.96^	0.96
2	SLR = 43.99H − 53.40	0.36	SLR = 6.71 H^1.48^	0.88
3	SLR = 40.60H + 26.29	0.31	SLR = 8.49H^1.50^	0.81
4	SLR = 37.70H − 11.31	0.25	SLR = 6.50 H^1.39^	0.72
5	SLR = 8.54H + 8.33	0.18	SLR = 5.75H^0.93^	0.93
6	SLR = 1.841H +0.86	0.10	SLR = 0.67 H^0.91^	0.89
7	SLR = 1.42H − 0.16	0.60	SLR = 0.76 H^0.94^	0.96
8	SLR = 1.27H − 0.08	0.47	SLR = 0.68 H^0.92^	0.96
9	SLR = 15.37H − 6.89	0.46	SLR = 2.15 H^1.17^	0.92

Note: R^2^ = determinant coefficient.

**Table 8:** Summary results of the general linear model (GML) for event soil loss rate as the target variable.

GLM	*SS*	*Df*	*F*	*P*	ŋ^2^p
Model	21,444,984	14	30.9	<0.001	0.369
Slope degree	3,800,420	5	60.3	<0.001	0.094
Soil cover	8,769,706	8	17.4	<0.001	0.193
Rainfall erosivity	8,661,173	1	137.5	<0.001	0.191
Rainfall duration	213,682	1	3.4	<0.001	0.006
Slope degree × Soil cover	0.00	40	-	-	0.000
Residuals	36,726,898	583			
Total	58,171,881	597			

Note: *SS* represents sum of squares, *Df* represents the degrees of freedom, *F* represents the statistic value, *p* represents significance, and ŋ^2^p represents the effect size.
